# Revealed: The spy who regulates neuroblastoma stem cells

**DOI:** 10.18632/oncotarget.2839

**Published:** 2014-12-02

**Authors:** Parvez Vora, Chitra Venugopal, Sheila K. Singh

**Affiliations:** ^1^ McMaster Stem Cell and Cancer Research Institute, McMaster University, Hamilton, Canada; ^2^ Department of Biochemistry and Biomedical Sciences, Faculty of Health Sciences, McMaster University, Hamilton, Canada; ^3^ Department of Surgery, Faculty of Health Sciences, McMaster University, Hamilton, Canada

**Keywords:** Spy1, neuroblastoma, CD133

Neuroblastoma (NB), an embryonal tumour of the sympathetic nervous system, is thought to originate from undifferentiated neural crest cells and is known to exhibit extremely heterogeneous biological and clinical behaviors. Occurring in very young children, the median age at diagnosis is 17 months and it accounts for 10% of all pediatric cancer mortalities [1, 2]. The standard treatment regimen for patients with high-risk NB includes induction and surgery followed by isotretinoin or Accutane (13-cis retinoic acid) treatment, which is shown to induce terminal differentiation of NB cells [3]. However, molecular regulators that maintain an undifferentiated phenotype in NB cells are still poorly understood.

Human cancer cells can arise from either stem/progenitor cells that fail to exit the cell cycle and differentiate, or from de-differentiated cells that have re-entered the cell cycle. A pre-requisite for differentiation is induction of cell cycle exit by the cell cycle regulators. One such cell cycle regulator, Speedy/RINGO (Spy1), was recently shown to maintain glioma tumour initiating cells (TICs) by regulating cell proliferation and stemness [4]. Spy1, an activator of cyclin-dependant kinases (CDK), is known to mediate cell cycle progression and cell survival in response to DNA damage [5, 6]. In the recent issue of *Oncoscience*, researchers give a mechanistic insight into Spy1-mediated regulation of NB stem cell differentiation [7].

Interestingly, when human neuroblastoma SH-SY5Y cells were subjected to 13-*cis*-Retinoic Acid (RA)-induced differentiation, the endogenous transcript as well as protein levels of Spy1 deplete significantly. Moreover, Spy1 expression level was shown to correlate with decreased levels of neural stem cell maker Nestin and increased levels of neuronal differentiation marker GAP43. The kinase activity of CDK2, a downstream signaling mediator of Spy1, was also shown to decline concurrently with Spy1 expression.

**Figure 1:**
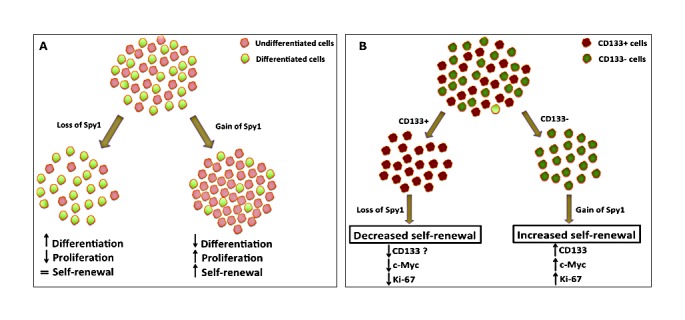
Spy1 regulates Neuroblastoma (NB) stem cells (A) Upon RA-induced differentiation, Spy1 depletion caused decreased proliferation and increased differentiation, while Spy1 overexpression delayed differentiation and promoted proliferation in NB cell lines. (B) Knockdown of Spy1 in CD133+ NB stem cells resulted in decreased clonogenicity. When Spy1 was overexpressed in the CD133− NB subpopulation, there was a significant rise in the expression of stem cell markers and showed increased self-renewal property.

Furthermore, using both shRNA knockdown and overexpression approaches, the authors demonstrate a significant functional contribution of Spy1 to self-renewal, proliferation and differentiation of NB cell lines (Figure 1a). Spy1 overexpressing cell lines failed to differentiate upon RA induction, continued proliferating and demonstrated enhanced self-renewal ability compared to the control cell line. Conversely, loss of Spy1 in NB cell lines lead to decreased cell proliferation and induced neuronal differentiation. The effect on Spy1 gain/loss of function in NB cells must be evaluated on *in vivo* tumour-forming mouse models before translating these exciting findings to the next level.

Numerous studies have shown that stemness in cancer is maintained in a hypoxic microenvironment. Growing NB cell lines as spheroid suspension cultures lead to increased expression of stem cell markers *Oct4* and *Bmi1* in Spy1-elevated cells. Providing a brief insight into the role of micro-environmental conditions and Spy1 functionality in regulating stemness, the authors underscore the importance of spheroids as 3D models of *in vivo* solid tumours.

The authors then go on to delineate the mechanism of Spy1 action on putative NB TICs, marked by CD133 positivity. A previous study reported that the intermediate type (I-type) NB cells have high expression of CD133 compared to other less tumorigenic subtypes of NB [8]. However, a later study failed to detect CD133 expression in NB TICs [9]. The authors here show no difference in self-renewal ability of CD133+ vs CD133− NB cells. Thus, it remains unclear whether CD133− expressing NB cells possess the defining properties of TICs. Nevertheless, CD133 is shown to mechanistically play a role in regulating NB tumorigenesis and proliferation by preventing differentiation [10]. Here, the authors selectively knock down Spy1 in CD133+ NB cells and show a significant decline in sphere formation. But when Spy1 was overexpressed, the CD133− population showed enhanced self-renewal capacity (Figure 1b), accompanied by an increase in CD133 transcript levels. Is Spy1 indirectly regulating CD133 expression, which then blocks the differentiation of NB stem cells? These findings also suggest that Spy1 regulates clonogenicity of NB cells, possibly having a differential effect on CD133+ and CD133− populations. These findings could be substantiated by comparing the effects of Spy1 knockdown and overexpression in CD133+ and CD133− NB cell populations in parallel.

This research takes a step towards understanding the molecular regulation of NB TICs differentiation, the currently employed strategy to treat this dreadful tumour. Previous studies have shown that widespread differentiated NB tumours have a better prognosis than poorly differentiated ones [11, 12], an observation consistent with the CSC model in suggesting that undifferentiated NB cells drive tumour progression. This novel finding that Spy1, an atypical G1 phase regulator, is also a key suppresser of differentiation and a promoter of self-renewal of NB TICs, may yield more potent prognostic factors and druggable therapeutic targets for NB patients. Perhaps inhibition of Spy1 or its downstream signaling targets could contribute to less aggressive tumours and maybe even lead to new and improved therapies for high-risk NB patients in the clinic.
